# The El Niño Southern Oscillation and the salinity of land and water in the United States

**DOI:** 10.1371/journal.pone.0311544

**Published:** 2025-02-03

**Authors:** Nicola Limodio, Filippo De Marco, Ilaria Dal Barco

**Affiliations:** 1 Department of Finance, Bocconi University, Milan, Italy; 2 BAFFI, Bocconi University, Milan, Italy; 3 IGIER, Bocconi University, Milan, Italy; 4 CEPR, London, United Kingdom; Universidade de Aveiro, PORTUGAL

## Abstract

The El Niño–Southern Oscillation (ENSO) is a periodic climate phenomenon with important consequences for weather and socioeconomic variables worldwide. This paper investigates the effect of El Niño on the concentration of salts in soils and fresh water. Our analysis shows effects that are statistically significant and large in magnitude. In particular, in response to El Niño events, soil salinity increases by 21% in the northern region of the United States, where average temperatures are positively affected, while it decreases by 29% on average in the southern tiers of the country, where temperatures decline. Similarly, fresh water salinity increases by 9% in warmer counties, while it is 53% lower in cooler counties.

## Author summary

The El Niño–Southern Oscillation is a periodic climatic phenomenon, which alters weather patterns worldwide [1], causing floods [2], droughts [3] and extreme events [4–8]. It can damage ecosystems, disrupt oceanic life [9–12] and impact economies [13–17] and societies [18]. This study investigates El Niño’s impact on inland salinity levels in the United States, an area that shows a high degree of geographical heterogeneity in alterations of average and variability of temperatures. Through regression analysis, this study uncovers El Niño’s role in altering soil and river saltiness. Results indicate increased average salinity in warmer, drier regions, contrasting with decreased salinity in cooler, wetter areas. These findings elucidate the complex interplay between El Niño dynamics, temperature, precipitation, and salinity in terrestrial ecosystems. The relevance of these results is rooted in the importance of salinity levels for ecosystem’s health. Excess soil and freshwater salt disrupt plant root osmotic balance, impeding water and nutrient absorption. This hinders crop growth, leading to yield loss and potential crop failure [19]. Soil salinity also drives land degradation and desertification [20], posing economic losses and food security threats in affected regions.

## Introduction

The El Niño-Southern Oscillation (ENSO), commonly referred to as El Niño, is a periodic climatic phenomenon with intense local and global effects. Its occurrence is determined by changes in wind circulation patterns over the Pacific Ocean and it is the predominant source of the year-to-year variability of Earth’s climate [1]. ENSO events are complex phenomena, difficult to predict, and often influenced by other sources of atmospheric variability over the Pacific such as the North Pacific meridional mode (PMM) [21], the Aleutian Low (AL) [22], the North Pacific Oscillation (NPO) [23], or the Arctic Oscillation (AO) [24].

The occurrence of an El Niño event significantly alters weather patterns by significantly affecting average temperatures [4] and bringing heightened rainfall in some regions and severe droughts in others [3, 25]. In addition, it influences the likelihood of extreme weather events such as hurricanes and tropical cyclones [5, 6]. This climatic disruption, with its far-reaching consequences, leads to an amplified risk of wildfires [7], damages the tropical forest [8], influences vegetation growth in the Amazon basin [26], increases haze pollution [27], and jeopardizes crop yields in affected areas [13]. Moreover, El Niño can disrupt oceanic ecosystems, causing the dispersal of marine organisms [9] and the bleaching of coral reefs [10, 11], posing a serious threat to these delicate ecosystems. It has also been found responsible for changes in shorelines [12], coastal erosion, and flooding [2]. Beyond its environmental repercussions, it has been demonstrated that El Niño undermines economic development [14, 15], exerts a significant influence on the prices of both energy and non-fuel commodities [16] and affects banks, mortgages, and house prices [17]. Furthermore, it has been associated with an elevated probability of civil conflicts [18], further underscoring its multi-faceted impact on societies and economies worldwide.

In this study, we want to further investigate the repercussions of El Niño on the natural environment through an examination of its impacts on the salinity levels of soils and fresh water bodies in the United States. Although salinity levels in relation to ENSO events have already been explored, previous studies mainly focused on the salinity of the ocean. In particular, it has been shown that ocean levels of salinity are influenced by the ENSO cycle [28], but at the same time, salinity anomalies may also play an active role in ENSO evolution [29]. Our study examines instead the impact of the ENSO cycle on inland salinity levels. Using regression analysis, we present evidence that the occurrence of an ENSO event, by altering temperature and precipitation patterns, induces a substantial change in the saltiness of soils and rivers in the mainland United States. Specifically, consistent with the functioning of the water cycle, regions experiencing higher temperatures and reduced precipitations exhibit increased average salinity levels, while in regions where temperatures drop and precipitations are more abundant, average salinity is decreased.

This induced variation in salinity levels is particularly relevant because it affects ecosystems’ health. Excess salt in the soil and fresh water interferes with the osmotic balance of plant roots, limiting their ability to absorb water and essential nutrients. This can hinder the growth of many crop plants, reducing yields and, in severe cases, leading to crop failure. Different crops have varying levels of tolerance to salinity: above a specific threshold, which is characteristic for each variety of plant, yields start decreasing linearly [19]. Additionally, soil salinity is a prominent driver of land degradation and desertification [20], and it can render arable land unproductive and unsuitable for agriculture. This poses a significant threat of economic losses for farmers and may lead to food security issues in affected regions.

## Materials and methods

**Fig 1:**
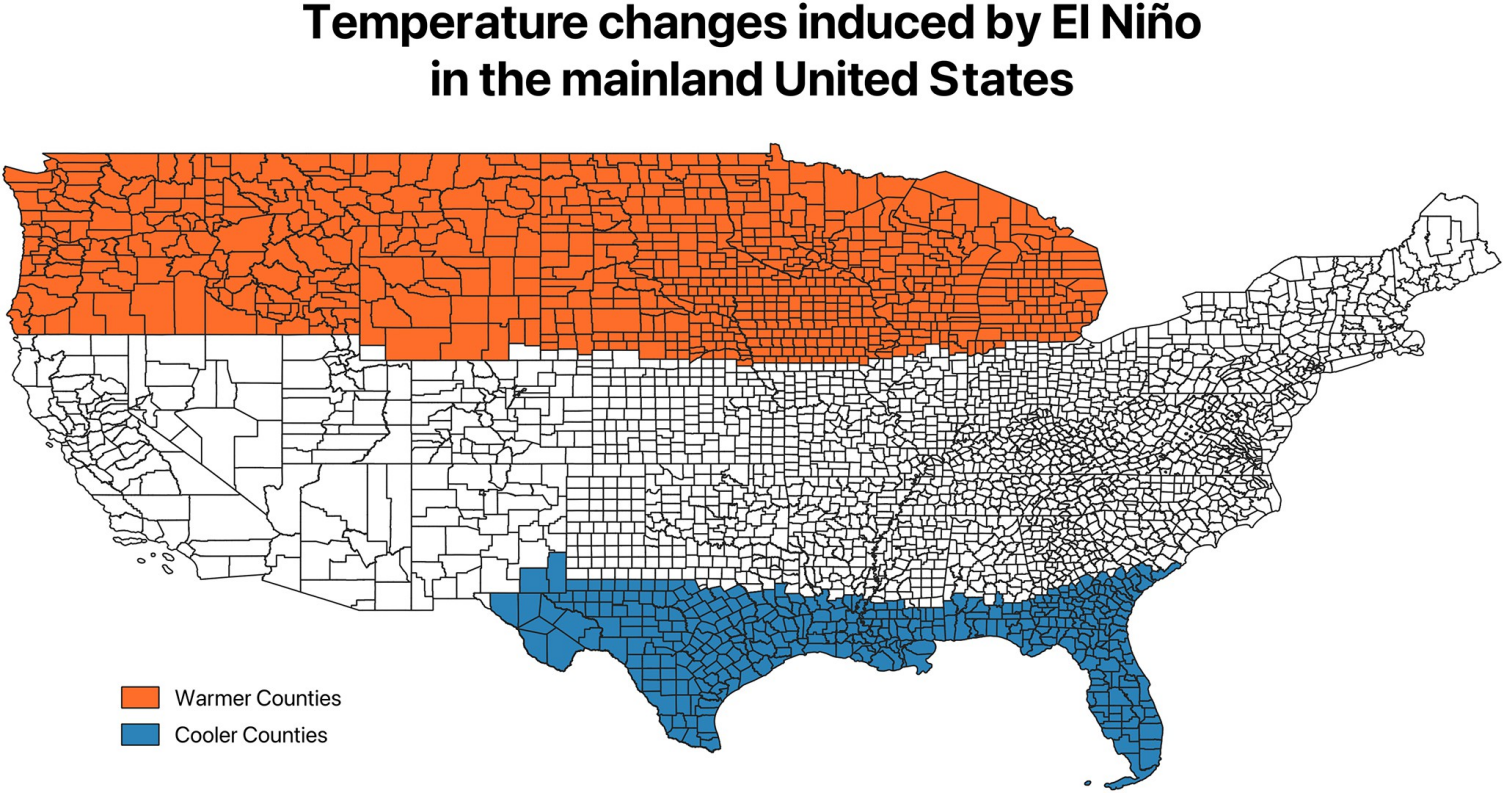
Geographical configuration of the temperature deviations from the mean during an El Niño event. Counties in orange experience a temperature increase, while the counties in blue are generally cooler during an El Niño episode. Affected counties are classified based on information gathered from the National Oceanic and Atmospheric Administration (NOAA).

For this analysis we used four sources of data:

1. Exposure to El Niño: To gather information on the geographical heterogeneity of ENSO impacts in the United States, we use a map elaborated by the National Oceanic and Atmospheric Administration (NOAA) (S3 Fig). This map uses color coding to show areas of warmer (orange) or colder (blue) temperatures and wetter (green) or drier (purple) conditions. By overlaying this map with the US county shapefile, we generate Fig 1, which illustrates the effects of El Niño on temperatures. Specifically, orange-shaded counties exhibit an increase in average temperatures, blue-shaded counties indicate a decrease, and white counties are neutral, showing no significant temperature change associated with El Niño events.
2. Occurrence of El Niño: There exist a variety of possibilities for measuring El Niño because it affects many elements of the atmosphere-ocean climate system. For this analysis, we employed the Multivariate ENSO Index Version 2 (MEI.v2), calculated as the leading principal component time series of the Empirical Orthogonal Function of five variables: sea level pressure (SLP), sea surface temperature (SST), surface zonal winds (U), surface meridional winds (V), and Outgoing Longwave Radiation (OLR). Values of MEI are produced for 12 partially overlapping 2-month “seasons”; here we take the average of the MEI index from January to May and define an El Niño year if this average is above a threshold of +1 degree Celsius (S1 Fig).
3. Soil Salinity: A recent assessment of soil salinity data has resulted in the “Global Soil Salinity Map” [30], obtained through the combination of soil properties maps with thermal infrared imagery and field observations. Using a machine learning framework, salinity maps were generated for seven selected years (1986, 1992, 2000, 2002, 2005, 2009, and 2016). Notably, two of these years (1992 and 2016) are classified as El Niño years in our framework, which allows a proper identification.
4. Water Salinity: We exploit a global harmonized database [31], created by combining electrical conductivity data from a range of different sources. Data are presented as measurements from monitoring stations in rivers, lakes, and groundwater in micro-Siemens per centimeter.

To achieve the identification of our results, we exploit the time variations of El Niño episodes and the geographical heterogeneity of El Niño in the United States. We use a Fixed Effects regression model, where the dependent variables are water and soil salinity. Counties and year Fixed Effects are introduced to capture possible time trends and characteristics that are specific of some counties/years, and that may correlate with El Niño. Additionally, to account for autocorrelation, we cluster standard errors at the county level.

## Results and discussion

### What is El Niño?

The El Niño Southern Oscillation is a natural cycle that fluctuates between a neutral and two extreme states, El Niño and La Niña, which are referred to as the cycle’s “positive” and “negative” phases, respectively.

During El Niño “neutral” years, trade winds on the Pacific Ocean blow towards west along the equator, taking warm water from South America towards Asia. These winds accumulate warm surface water near the Indonesian coast, raising sea levels there by nearly half a metre compared to the west coast of Latin America. As warm water is pushed towards west, the thermocline—the layer of water that separates surface waters from deep waters—slopes up from Asia towards South America. The thermocline divides the upper mixed layer, where water is warmer and temperature varies according to the season, from the calm deep water, where temperature is constant throughout the year because it is too deep to be influenced by solar radiation. In the eastern region of the Pacific, cold and nutrient-rich water emerges from the depths of the ocean, in a process known as upwelling. This supports high levels of primary productivity and diverse marine ecosystems. As a result, the sea surface temperature (SST) around the Asian coast is about 8°C warmer than in the eastern Pacific, and clouds and rainfall are more abundant near Asia and Australia, whereas the eastern Pacific remains relatively dry.

During El Niño, the trade winds are weakened and less warm water is pushed towards west. The thermocline is therefore flattened and the process of upwelling reduces its efficiency. Surface waters in the eastern Pacific are warmer and the supply of nutrients to the euphotic zone—the upper layers of a body of water into which sufficient light penetrates to permit the growth of green plants—is diminished. Rainfall follows the warm water eastward, causing flooding in Peru and droughts in Indonesia and Australia. The result is a great alteration of the global atmospheric circulation, which in turn affects weather patterns even in areas far from the tropical Pacific.

During La Niña, the situation is approximately reversed. Trade winds that push warm water toward west are stronger, resulting in a steeper inclination of the thermocline. The process of upwelling increases, bringing more cold and nutrient-rich water to the surface. The Eastern Pacific is cooler than usual, and the so-called cold tongue (the portion of cooler waters close to the South American west coast) extends farther westward.

This oscillation between states (positive, neutral, or negative) is characterized by great variation and uncertainty. Occurrences of El Niño events (positive states) range within a window of 2 to 7 years and are predicted with difficulty. They are determined considering the deviations from the mean of disparate oceanic and atmospheric conditions, including sea level pressure (SLP), sea surface temperature (SST), zonal and meridional components of the surface wind, and outgoing longwave radiation (OLR). By combining these variables, it is possible to obtain the Multivariate ENSO Index (MEI), an useful indicator able to capture the continuum of ENSO states.

### El Niño in the United States

The massive modifications in wind circulation patterns and ocean dynamics associated with the ENSO oscillation entail major implications for weather patterns worldwide. More specifically, in recent decades it has become clear that ENSO can manifest in two different types: the eastern Pacific (EP) type and central Pacific (CP) type. The EP type has its center of action in the equatorial eastern Pacific, typically associated with strong El Niño events, while the CP type has its center of action in the equatorial central pacific, often linked to strong La Niña events. These 2 phenomena can have markedly different effects on global temperature and precipitation patterns. Our study, however, specifically focuses on the mainland United States, which provides an ideal setting for our identification strategy. The reason is to be found in the high degree of geographical heterogeneity with respect to the alterations in average and variability of temperatures. Across the country, counties can be categorized into three groups based on whether they experience an increase, a decrease, or no change in average temperatures. Among the two ENSO types, the EP type has the most substantial impact on the U.S., leading to more pronounced changes in temperature and precipitation averages [32]. Consequently, the effects on inland salinity levels in the United States are more significant during EP-type events, and our analysis is therefore concentrated on these.

The most relevant changes induced by the EP-type ENSO in North America are due to the shifting of the paths of the jet streams. Jet streams are fast and narrow air currents that flow west to east. On planet Earth, there exist two main types of jet streams: polar jets and subtropical jets. Both the Northern and Southern Hemispheres have a polar jet and a subtropical jet. During El Niño, the subtropical jet stream, with its huge amounts of tropical moisture, is enhanced and shifted towards California, south of its normal position. As a consequence, the southern part of the nation experiences cooler and wetter than normal conditions, whereas the northern states become warmer. Additionally, the mid-west is generally drier, as the region is particularly well excluded from the path of the jet stream.

During La Niña, these deviations from the average are approximately (but not exactly) reversed. The subtropical jet stream meanders north enough to increase precipitation in the Northwestern and Midwestern states. Additionally, the polar jet stream is stronger than normal, decreasing temperatures across the northern regions of the country. On the contrary, the southern tiers of the United States are left hotter and drier.

Despite the many similarities, the life cycles of El Niño and La Niña differ in many aspects [33]. In particular, El Niño events are often stronger than La Niña events [34]. For this reason, our analysis focuses on the occurrences of El Niño events, as their effects are expected to be more pronounced. Fig 1 illustrates the counties in the mainland U.S. affected by temperature changes during El Niño events. Specifically, the orange-shaded counties represent areas experiencing an increase in average temperatures, the blue-shaded counties indicate a decrease in average temperatures, and the white counties are those considered neutral, meaning they show no significant temperature change associated with El Niño events.

### Salinity, temperatures and precipitations

Salinity, sometimes also denoted as saltiness, is the term used to refer to the amount of salt dissolved in a body of water or to the salt content of the soil. This crucial environmental parameter is primarily regulated through the dynamics of the water cycle, as the main determinants of salinity can be identified with the natural processes of evaporation, temperature fluctuations, and precipitation frequency.

In the ocean, sea surface salinity (SSS) is mostly determined by the amount of moisture that gets drawn into the atmosphere. As winds blow over the ocean, water undergoes evaporation, transitioning from liquid to vapor. This process leaves behind salts, thereby increasing the salinity of the sea surface. Contrarily, when rain falls over the ocean, the sea surface water is diluted and becomes less salty. Typically, about 90 percent of the evaporated water returns to the ocean in the form of precipitation, while the remaining 10 percent is carried over land, where it finally turns into rainfall or snowfall. Following these dynamics, some studies have demonstrated a connection between the saltiness of the ocean and the amount of inland precipitations. If a portion of the ocean exhibits a higher than usual salinity, it indicates that there will be more rain in another area of the planet. Similarly, lower levels of ocean salinity imply that the ocean has retained more water and fewer precipitations will fall on land [35, 36].

Similarly to what happens in the ocean, precipitations on land are a crucial determinant of salinity levels. In areas with abundant precipitations, large amounts of water constantly infiltrate the soil, creating a sort of flushing effect that washes the salt away through streams and rivers. Wet areas, therefore, generally have a lower salt concentration. Conversely, in dry areas where rainfalls are rare, a greater share of the water that falls is lost through evaporation. Since water vapor does not contain any salt, it is entirely left in the soil, where it accumulates over time, increasing the salinity of the soil and groundwater. Temperatures, by influencing the rate and pace of water evaporation, are also crucial. Warmer zones experience more evaporation and, consequently, higher salinity concentrations, while colder areas remain generally more humid.

### Salinity and El Niño

The ENSO oscillation, by changing patterns of temperature and precipitation, has the potential to influence salinity levels. In the past decades, substantial evidence has been produced that links El Niño with salinity alterations in the Pacific Ocean [37–41]. Specifically, research shows that the tropical Pacific, and in particular the western Pacific warm pool, exhibit what are referred to as Sea Surface Salinity Anomalies (SSSA). These episodes are characterized by significant deviations from the average sea surface salinity values and are considered an indication of the development of an El Niño event [42]. Additionally, evidence suggests that, beyond the passive response, salinity variability may also actively contribute to ENSO evolution [43] and is thus important for the forecasting of El Niño events [29], and for the study of ENSO dynamics [44].

The main novelty of this study is the establishment of a relationship between El Niño events and the salt content of soil and fresh water. The identification is achieved by exploiting simultaneously the geographical heterogeneity of ENSO impacts across the United States and the time fluctuation of El Niño. For the cross-sectional variation, we adopt a map elaborated by climatologists of the National Oceanic and Atmospheric Administration (NOAA), from which we are able to classify each county as either being affected by warmer-than-average temperatures, cooler-than-average temperatures, or unaffected, as shown in Fig 1. To account for time variations, since there is a continuum of El Niño conditions, we classify as positive El Niño years the 5 strongest events since 1980, i.e. those years with an increase in average oceanic temperatures greater than one degree Celsius (S1 Fig).

To quantify the effects of temperature variations induced by El Niño on salinity levels, we employ the following specification:

$$Y_{cy}=\alpha_{c}+\gamma_{y}+\beta Exposure_{c}\times ElNi\tilde{n}o_{y}+\epsilon_{cy}$$

where *Y*_*ct*_ is the salinity of soil or fresh waters in county *c* in year *y*, *α*_*c*_ and *γ*_*y*_ are county and year Fixed Effects, *Exposure*_*c*_ represents whether a county is warmer, cooler, or unaffected by the presence of El Niño and *ElNiño_y_* is a dummy variable that takes unit value in case of a top 5 El Niño event.

The results of the analysis are in line with our hypothesis: El Niño, despite being a temporary phenomenon, induces weather shocks with the capability of altering the salt content of soil and fresh waters. In particular, during an El Niño event, in warmer counties the level of soil salinity is 22% higher than the mean, while in cooler counties the level of soil salinity is 28% lower, with all these figures being significant below the 1% threshold (Table 1).

**Table 1:** Effects of El Niño-induced changes in temperature on inland salinity.

Variables	(1)	(2)	(3)	(4)	(5)	(6)
	Soil Salinity			Water Salinity		
*Warmer*_*c*_ × *El Nino*_*y*_	0.0416*** (0.00411)		0.0347*** (0.00420)	0.125*** (0.0290)		0.0682*** (0.0260)
*Cooler*_*c*_ × *El Nino*_*y*_		-0.0547*** (0.00761)	-0.0455*** (0.00778)		-0.407*** (0.119)	-0.388*** (0.120)
County FE	Yes	Yes	Yes	Yes	Yes	Yes
Year FE	Yes	Yes	Yes	Yes	Yes	Yes
Obs.	21756	21756	21756	56514	56514	56514
Adj. R sq.	0.740	0.740	0.741	0.727	0.727	0.727
Mean Dep. Var.	0.160	0.160	0.160	1.047	1.047	1.047

The table presents ordinary least squares (OLS) estimates, where the unit of observation is county *c* in year *y*. County and year fixed effects are present in all columns, and standard errors are clustered at the county level. The dependent variable in the first 3 columns is the average indicator of soil salinity, while the dependent variable in columns 4 to 6 is the average value of water salinity, measured in microSiemens per centimetre $\frac{\mu S}{cm}$. *Warmer*_*c*_ and *Cooler*_*c*_ are dummy variables that take unit value if a county is exposed to an increase or a decrease in temperatures during an El Niño event, respectively, while *El Niño_y_* takes unit value if year *y* exhibits a top 5 El Niño event. Obs. refers to the number of observations, Adj. R sq. refers to the adjusted *R*^2^ and Mean Dep. Var. refers to the mean value of the dependent variable. ***, ** and * indicate significance at the 1%, 5% and 10% levels, respectively.

**Fig 2:**
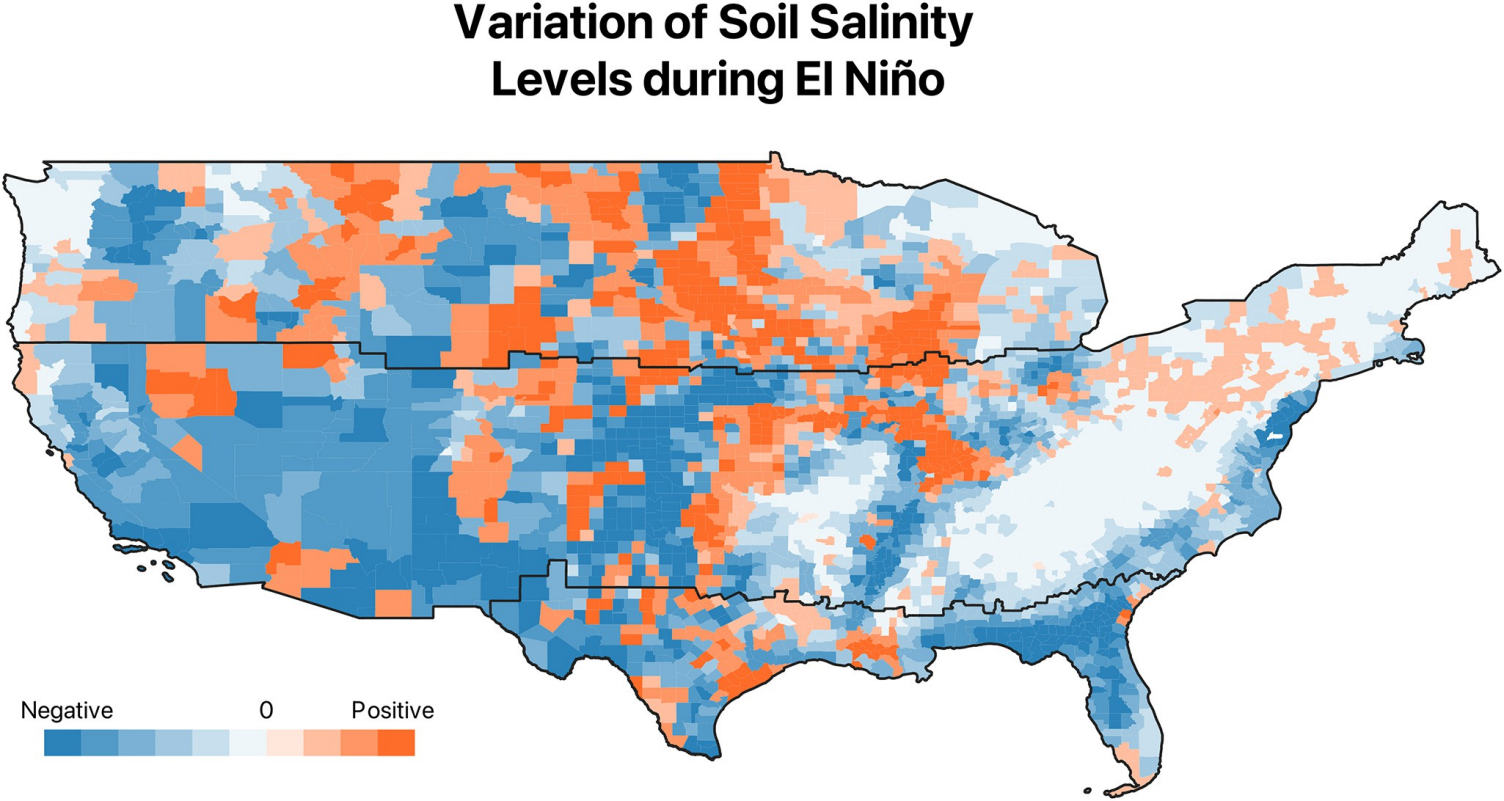
Difference between average soil salinity levels during El Niño years and neutral years. Orange stands for a positive difference between El Niño years and Neutral years, i.e. there is an increase of soil salinity levels, while blue denotes counties that exhibit a negative difference, i.e. soil salinity levels decline.

**Fig 3:**
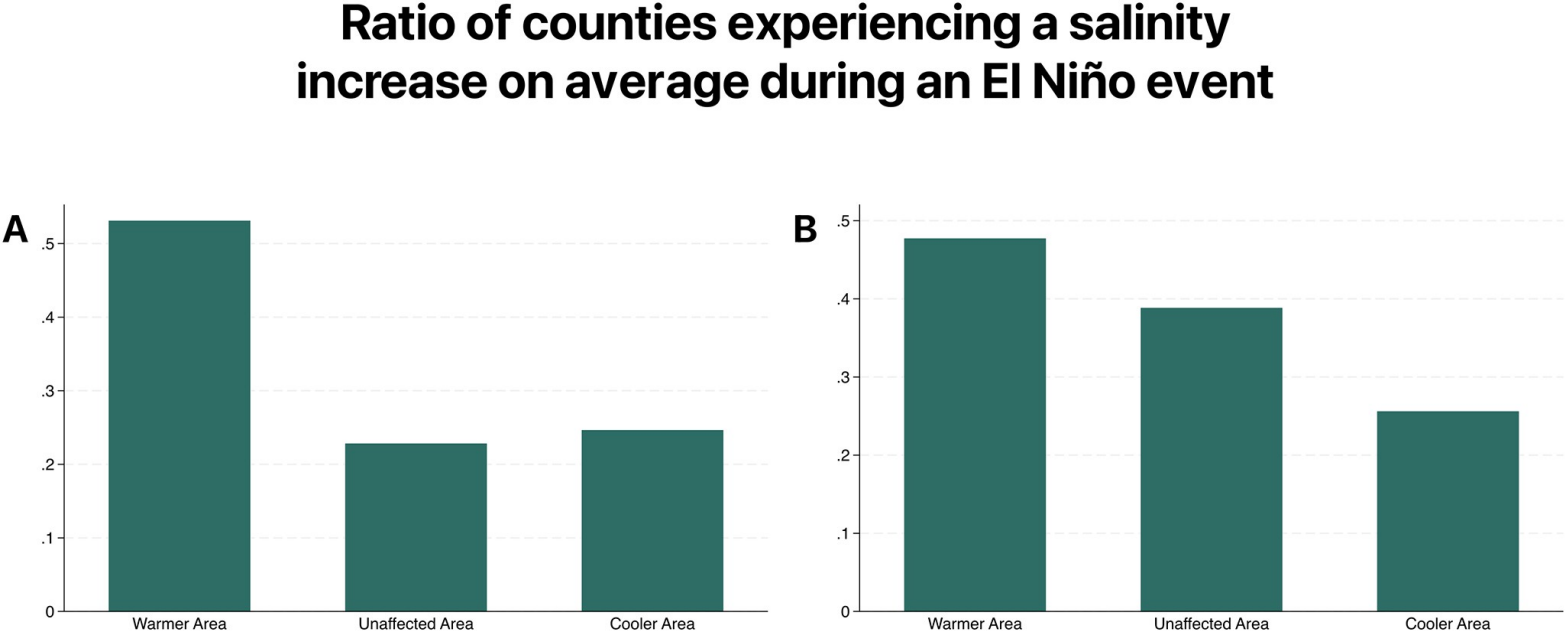
Proportion of counties that experience, on average, an increase in salinity levels during El Niño years, divided by geographical areas. A: Percentage of counties that experience an increase in soil salinity during El Niño is higher in counties with a positive deviation from mean temperatures. B: Percentage of counties that experience an increase in water salinity during El Niño is higher in counties with a positive deviation from mean temperatures.

Fig 2 presents a map depicting the average changes in soil salinity for each US county. Orange areas are those whose salinity levels are higher than average during El Niño years, while the blue color indicates a negative change. In the northern area, the proportion of orange counties is sensibly higher, if compared with the cooler or unaffected areas. This difference becomes even more apparent by looking at the left panel of Fig 3: among warmer counties, more than 53% experience an increase in soil salinity, while this is true only for 25% and 23% of cooler and unaffected counties, respectively.

The results of the analysis on inland fresh water bodies show the same patterns, with salinity levels of rivers being 7% higher in warmer counties and 37% lower in cooler counties (Table 1). Similar to before, the right panel of Fig 3 graphically depicts how the concentration of counties that experience an increase in salinity is higher in the warmer area (48%) rather than in the cooler (27%) or unaffected (39%) areas (The opposite is true for a decrease in salinity levels S2 Fig).

These results highlight that salinity levels react with high sensitivity to changes in average temperatures. The same is true for changes in the frequency of precipitations, as highlighted by S1 Table in the supplementary materials.

The mechanisms through which ENSO affects salinity in U.S. land and water bodies can be traced to the ENSO-induced changes in regional precipitation and temperature patterns. During El Niño events, increased rainfall in certain regions can boost freshwater inflow into rivers and lakes, with consequent dilution of salt concentration. Conversely, areas experiencing reduced rainfall may see an increase in salinity due to diminished freshwater input. Similarly, warmer temperatures typical of El Niño events can enhance evaporation rates, resulting in higher concentrations of salts in soils and inland water bodies. In contrast, cooler temperatures can reduce evaporation rates, helping to retain moisture and reduce salinity levels. Additionally, there may also be indirect mechanisms by which ENSO events can influence inland salinity, for instance through their impact on agricultural practices. Shifts in rainfall and temperature can alter irrigation demands, potentially leading to increased use of saline water sources or changes in irrigation practices that affect soil salinity.

## Conclusion

The analysis of the effects of the ENSO cycle has been escalating in the last couple of decades. This boost is justified by the intensification of the strength of El Niño episodes and by the magnitude of their effects on socioeconomic variables. In addition, in the context of a changing climate, the ENSO oscillation offers a unique setting to investigate the possible consequences of global warming and the rise of extreme weather events.

Our results show that the meteorological shocks caused by El Niño, even if transitory, have significant effects on the salt content of freshwater and soils. These findings confirm the major role that temperature plays in salinity levels and soil health, raising many concerns about the threats that global warming might pose to the health of soils, agricultural yields, and food security.

## Supporting information

S1 Fig Continuum of El Niño episodes. The y-axis measures the values of the MEI index, as an average between January and May. On the x-axis, there are years with a positive MEI. The 5 strongest El Niño years are highlighted by the horizontal dashed line.
(TIFF)

S2 Fig Proportion of counties that experience, on average, a decrease in salinity levels during El Niño years, divided by geographical areas. A: Percentage of counties that experience a decline in soil salinity during El Niño is higher in counties with a negative deviation from mean temperatures. B: Percentage of counties that experience a decline in water salinity during El Niño is higher in counties with a negative deviation from mean temperatures.
(TIFF)

S3 Fig El Niño effects in the US. Map elaborated by the National Oceanic and Atmospheric Administration (NOAA). Highlighted with different colors are regions that experience a change in temperatures or precipitation frequency during El Niño episodes.
(TIFF)

S1 Table Effects of El Niño-induced changes in precipitations on inland salinity. (PDF)
