# Supplementary figures and images for: The El Niño Southern Oscillation and the salinity of land and water in the United States
Source: PLoS One. 2025 Feb 3;20(2):e0311544. doi: 10.1371/journal.pone.0311544 (PMC11790109; doi:10.1371/journal.pone.0311544)

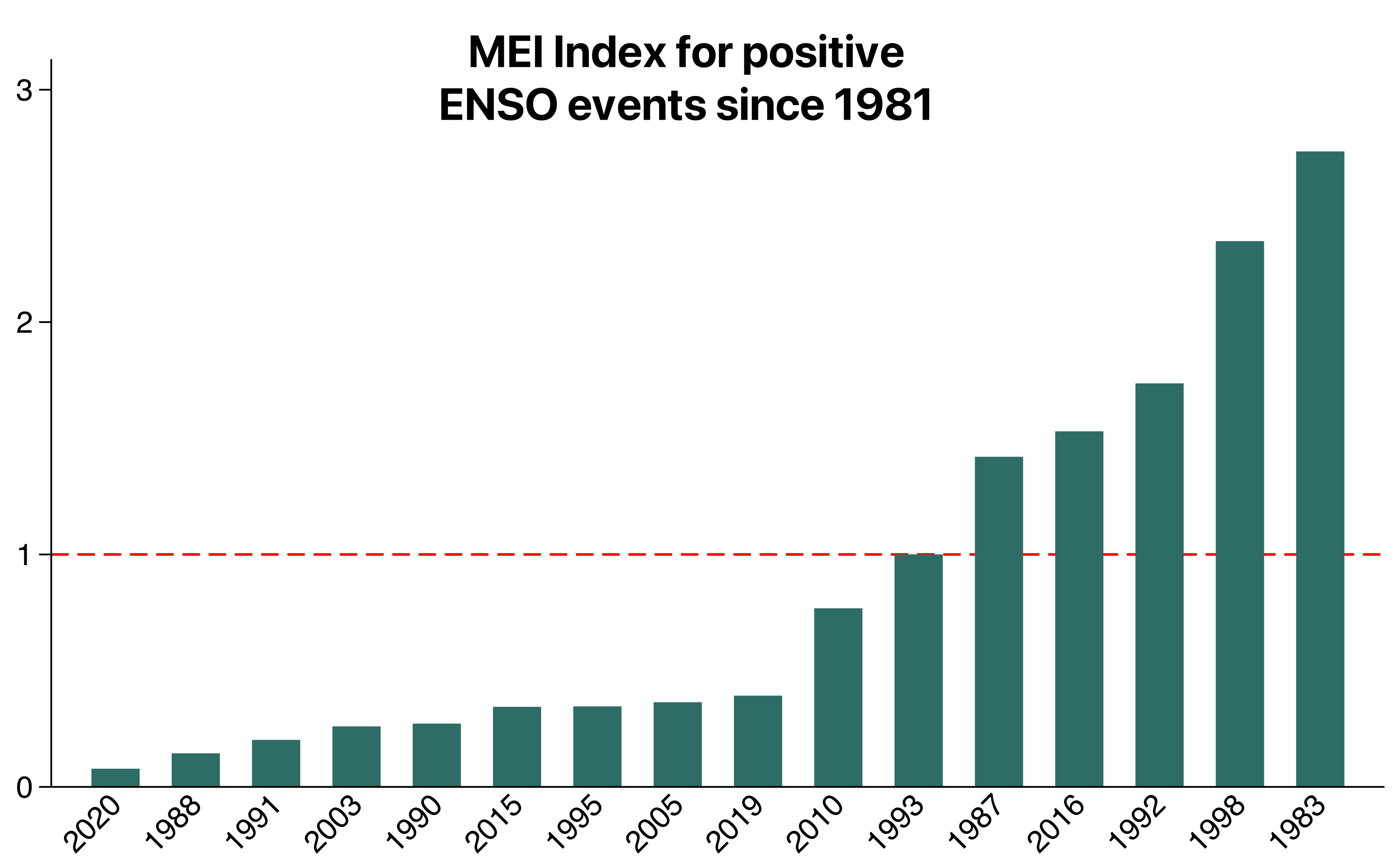

Supplement: S1 Fig — The y-axis measures the values of the MEI index, as an average between January and May. On the x-axis, there are years with a positive MEI. The 5 strongest El Niño years are highlighted by the horizontal dashed line. (TIFF) [file pone.0311544.s001.tiff]

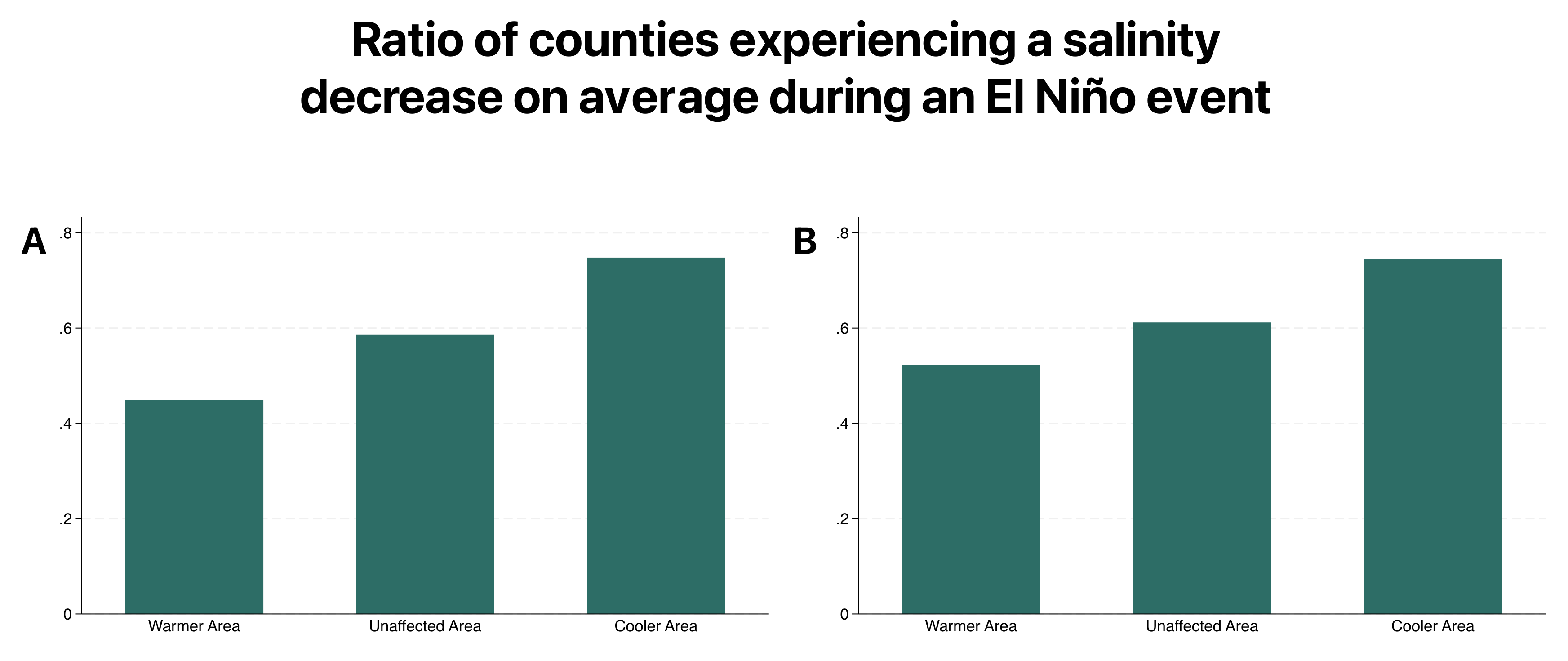

Supplement: S2 Fig — A: Percentage of counties that experience a decline in soil salinity during El Niño is higher in counties with a negative deviation from mean temperatures. B: Percentage of counties that experience a decline in water salinity during El Niño is higher in counties with a negative deviation from mean temperatures. (TIFF) [file pone.0311544.s002.tiff]

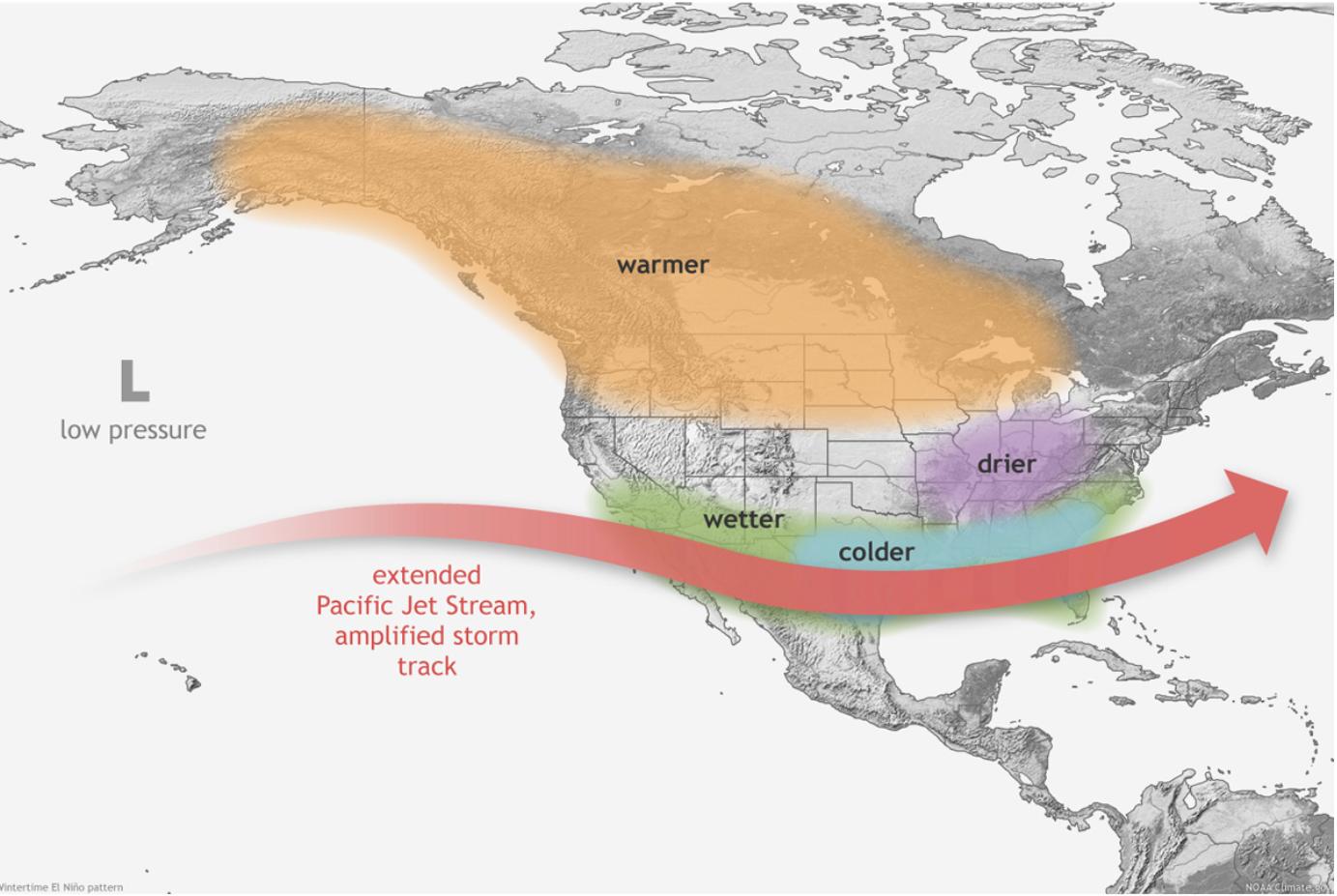

Supplement: S3 Fig — Map elaborated by the National Oceanic and Atmospheric Administration (NOAA). Highlighted with different colors are regions that experience a change in temperatures or precipitation frequency during El Niño episodes. (TIFF) [file pone.0311544.s003.tiff]
