# Supplementary material for: The El Niño Southern Oscillation and the salinity of land and water in the United States
Source: PLoS One. 2025 Feb 3;20(2):e0311544. doi: 10.1371/journal.pone.0311544 (PMC11790109; doi:10.1371/journal.pone.0311544)
Supplement: S1 Table — (PDF) [file pone.0311544.s004.pdf]

S1 Table. Effects of El Niño-induced changes in precipitations on inland salinity.

	(1)	(2)	(3)	(4)	(5)	(6)
Variables		Soil Salinity			Water Salinity	
$Wetter_c \times El\ Nino_y$	-0.0450***		-0.0356***	-0.313***		-0.303***
	((0.00537)		(0.00550)	(0.0789)		(0.0807)
$Drier_c \times El\ Nino_y$		0.0360***	0.0267***		0.109***	0.0328
		(0.00514)	(0.00529)		(0.0267)	(0.0241)
County FE	Yes	Yes	Yes	Yes	Yes	Yes
Year FE	Yes	Yes	Yes	Yes	Yes	Yes
Obs.	21756	21756	21756	56514	56514	56514
Adj. R sq.	0.740	0.740	0.741	0.727	0.727	0.727
Mean Dep. Var.	0.160	0.160	0.160	1.047	1.047	1.047

The table presents ordinary least squares (OLS) estimates, where the unit of observation is county c in year y. County and year fixed effects are present in all columns, and standard errors are clustered at the county level. The dependent variable in the first 3 columns is the average indicator of soil salinity, while the dependent variable in columns 4 to 6 is the average value of water salinity, measured in microSiemens per centimetre $\frac{\mu S}{cm}$. $Wetter_c$ and $Drier_c$ are dummy variables that take unit value if a county is exposed to an increase or a decrease in precipitations during an El Niño event, respectively, while $El\ Niño_y$ takes unit value if year y exhibits a top 5 El Niño event. Obs. refers to the number of observations, Adj. R sq. refers to the adjusted R^2 and Mean Dep. Var. refers to the mean value of the dependent variable. ***, ** and * indicate significance at the 1%, 5% and 10% levels, respectively.
